# Supplementary material for: Quantitative Characterization of the Impact of Protein–Protein Interactions on Ligand–Protein Binding: A Multi-Chain Dynamics Perturbation Analysis Method
Source: Int J Mol Sci. 2024 Aug 23;25(17):9172. doi: 10.3390/ijms25179172 (PMC11394879; doi:10.3390/ijms25179172)
Supplement: Supplementary file 1 [file ijms-25-09172-s001.zip › Table S1-PPI_effects_on_mcDPA.pdf]

Table S1 Changes in *mc*DPA predicted ligand binding regions upon introduction of protein-protein interactions

**Category**	**A**	**Chain**	**A+B**	**Chains**	**Change**	**Overlap**	
						**Precision**	**Recall**
OG	1QG4	A	1A2K	D/A	**O→O′**<br>**P→Ø**<br>**Ø→{P′, Q′}**	**0.57**	**0.32**
OG	1AZT	A	1AZS	C/B	**O→O′**<br>**{P,Q}→Ø**<br>**Ø→{P′, Q′}**	**0.91**	**0.91**
OG	1MH1	A	1E96	A/B	**O→Q′**<br>**Ø→{O′, P′}**	**0.70**	**0.83**
OG	1TND	A	1FQJ	A/B	**O→O′**<br>**O→P′**<br>**Q→Q′**<br>**P→Ø**<br>**Ø→R′**	**1.00**<br>**0.42**<br>**1.00**	**0.75**<br>**1.00**<br>**0.30**
OG	1GIA	A	1GP2	A/B	**O→Ø**<br>**P→R′**<br>**Ø→{O′,P′,Q′,S′,**<br>**T′,U′,V′,W′}**	 **0.91**	 **1.00**
OG	1A4R	A	1GRN	A/B	**O→O′**<br>**Ø→{P′, Q′}**	**0.75**	**0.51**
OG	1MH1	A	1HE1	C/A	**O→O′**<br>**Ø→{P′, Q′, R′}**	**1.00**	**0.45**
OG	821P	A	1HE8	B/A	**O→O′**<br>**Ø→{P′, Q′, R′, S′,**<br>**T′}**	**0.29**	**0.04**
OG	1MH1	A	1I4D	D/A	**O→P′**<br>**Ø→O′**	**0.43**	**1.00**
OG	1QG4	A	1IBR	A/B	**O→U′**<br>**P→Ø**<br>**Ø→{O′, P′, Q′, R′,**<br>**S′, T′}**	**0.86**	**0.15**
OG	1O3Y	A	1J2J	A/B	**O→R′**<br>**P→P′**<br>**Ø→{O′, Q′}**	**0.20**<br>**0.73**	**0.25**<br>**1.00**
OG	1RRP	A	1K5D	A/B	**O→O′**<br>**P→P′**	**0.20**<br>**0.43**	**0.88**<br>**1.00**
OG	5P21	A	1LFD	B/A	**O→O′**<br>**P→Q′**<br>**Ø→{P′, R′}**	**0.62**<br>**0.67**	**0.39**<br>**0.55**
OG	1HUR	A	1R8S	A/E	**O→O′**<br>**P→Ø**<br>**Q→Q′**<br>**Ø→P′**	**0.50**<br><br>**0.76**	**0.71**<br><br>**0.62**
OG	6Q21	A	1WQ1	R/G	**{O, P, Q}→Ø**<br>**Ø→{O′, P′, Q′, R′}**		
OG	2BME	A	1Z0K	A/B	**O→Ø**<br>**Ø→{O′, P′, Q′}**		
OG	1MH1	A	2FJU	A/B	**O→P′**<br>**Ø→{O′, Q′, R′, S′,**	**0.70**	**1.00**

					T′}		
OG	1Z06	A	2G77	B/A	O→Q′<br>P→Ø<br>Ø→{O′, P′, R′}	0.38	0.86
OG	1GFI	A	2GTP	A/D	O→P′<br>O→Q′<br>P→O′<br>Ø→R′	0.46<br>0.23<br>1.00	1.00<br>0.32<br>0.94
OG	1MH1	A	2H7V	A/C	O→O′<br>Ø→{P′, Q′, R′}	0.70	0.76
OG	1G16	A	3CPH	A/G	O→Ø<br>Ø→{O′, P′, Q′, R′, S′}		
OX	1IJJ	A	1ATN	A/D	O→O′<br>{P,Q}→Ø<br>R→Q′<br>Ø→{P′, R′, S′}	1.00<br><br>0.82	1.00<br><br>0.52
OX	3DNI	A	1ATN	D/A	O→R′<br>Ø→{O′, P′, S′}	0.95	1.00
OX	1QRQ	A	1EXB	A/E	O→Q′<br>O→S′<br>P→O′<br>Ø→{P′, R′}	0.42<br>0.19<br>0.96	1.00<br>1.00<br>0.97
OX	1IJJ	A	1H1V	A/G	{O,P,Q,R}→Ø<br>Ø→{O′, P′, Q′, R′, S′}		
OX	1KUY	A	1IB1	E/A	O→O′<br>P→Ø<br>Ø→{P′, Q′, R′}	0.82	1.00
OX	1IJJ	A	1KXP	A/D	O→O′<br>{P,Q}→Ø<br>R→P′	1.00<br><br>0.36	0.65<br><br>1.00
OX	1IAM	A	1MQ8	A/B	{O,P,Q}→Ø<br>Ø→{O′, P′, Q′}		
OX	3MIN	A	1N2C	A/B	O→P′<br>P→S′<br>Ø→{O′, Q′, R′}	0.28<br>0.44	1.00<br>1.00
OX	2VAW	A	1OFU	A/X	O→Q′<br>{P,Q}→Ø<br>R→R′<br>Ø→{O′, P′, S′}	0.92<br><br>1.00	1.00<br><br>1.00
OX	2FXU	A	1Y64	A/B	O→Ø<br>Ø→{O′, P′, Q′, R′, S′}		
OX	1IJJ	A	2BTF	A/P	O→O′<br>{P,Q,R}→Ø	0.92	0.40
OX	1NG1	A	2J7P	A/D	O→O′<br>O→P′<br>{P,Q}→Ø<br>Ø→{Q′, R′, S′}	0.44<br>0.38	1.00<br>0.50
OX	2IYL	D	2J7P	D/A	{O,P}→Ø<br>Ø→{O′, P′, Q′, R′, S′}		
OX	3BIX	A	3BIW	A/E	O→P′<br>P→Q′	0.89<br>1.00	1.00<br>1.00

					**{Q,R,T,U,W,X}→Ø**<br>**S→S′**<br>**V→R′**<br>**Ø→{O′, T′}**	 **0.94**<br>**1.00**	 **1.00**<br>**0.94**
OX	1IJJ	A	3DAW	A/B	**{O,Q}→Ø**<br>**P→P′**<br>**R→P′**<br>**Ø→O′**	 **0.40**<br>**0.82**	 **0.21**<br>**0.64**
OX	3ODQ	A	3SZK	D/F	**O→O′**<br>**Ø→P′**	**0.85**	**1.00**
ES	1E1N	A	1E6E	A/B	**{O,P,Q,S,T} →Ø**<br>**R→O′**<br>**R→P′**	 **0.94**<br>**0.82**	 **0.57**<br>**0.64**
ES	1CL0	A	1F6M	A/C	**O→O′**<br>**Ø→P′**	**0.89**	**0.30**
ES	1B39	A	1FQ1	B/A	**O→O′**<br>**P→O′**<br>**Ø→{P′, R′}**	**0.44**<br>**0.14**	**0.17**<br>**0.01**
ES	1XK9	A	1ZM4	B/A	**O→Q′**<br>**O→R′**<br>**Ø→{O′, P′, S′, T′, U′, V′, W′, X′}**	**0.90**<br>**0.70**	**0.88**<br>**1.00**
ES	1U90	A	2A9K	A/B	**{O,P,Q}→Ø**<br>**Ø→O′**		
ES	1J54	A	2IDO	A/B	**O→P′**<br>**Ø→O′**	**0.93**	**1.00**
ES	1CCP	A	2PCC	A/B	**O→P′**<br>**P→Q′**<br>**Ø→{O′, R′, S′, T′}**	**0.88**<br>**1.00**	**1.00**<br>**1.00**
ES	1YCC	A	2PCC	B/A	**O→P′**<br>**{P,Q}→Ø**<br>**Ø→{O′, Q′, R′, S′, T′}**	**0.04**	**0.15**
ES	1GIQ	A	4H03	A/B	**O→P′**<br>**O→U′**<br>**P→Q′**<br>**P→R′**<br>**Q→S′**<br>**R→U′**<br>**Ø→{O′, T′}**	**0.91**<br>**0.12**<br>**0.27**<br>**0.75**<br>**0.58**<br>**1.00**	**1.00**<br>**0.10**<br>**1.00**<br>**0.79**<br>**1.00**<br>**1.00**
ES	1IJJ	A	4H03	B/A	**{O,P,Q,R}→Ø**<br>**Ø→{O′, P′, Q′, R′, S′ T′, U′}**		
ER	1JMJ	A	1JMO	A/H	**O→O′**<br>**P→Ø**<br>**Ø→{ P′, Q′, R′}**	**0.76**	**0.80**
ER	2CN0	H	1JMO	H/A	**O→O′**<br>**P→Ø**<br>**Ø→{P′, Q′, R′}**	**0.29**	**0.23**
ER	3C13	A	1JWH	A/C	**O→P′**<br>**P→O′**<br>**{Q,R,S}→Ø**<br>**Ø→Q′**	**0.56**<br>**0.50**	**0.53**<br>**0.16**
ER	1V8Z	A	1WDW	B/A	**O→Ø**<br>**P→O′**	 **0.40**	 **0.04**

					Q→O′<br>Ø→{P′, Q′}	1.00	0.28
ER	1E3T	A	2OOR	C/A	O→Ø<br>Ø→{O′, P′, Q′}		
ER	1L7E	A	2OOR	A/C	O→O′<br>Ø→{ P′, Q′}	0.95	0.36
ER	2YVF	A	2YVJ	A/B	{O,R}→Ø<br>P→Q′<br>Q→S′<br>Ø→{P′, R′, T′, U′}	 0.87<br>0.76	 0.85<br>1.00
AA	1HRC	A	1WEJ	F/H	O→O′<br>P→Ø<br>Ø→{P′, Q′, R′}	0.78	1.00
AA	1YWH	A	2FD6	U/A	O→Q′<br>P→P′<br>P→Q′<br>Ø→{R′, S′}	0.75<br>0.27<br>0.14	0.13<br>0.48<br>0.13
AA	3TGT	A	3SE8	G/H	O→P′<br>P→Ø<br>Q→V′<br>R→Q′<br>Ø→{O′, R′, S′, T′, U′}	0.90<br><br>0.83<br>1.00	1.00<br><br>0.83<br>0.81
AA	3TGT	A	3U7Y	G/H	O→S′<br>{P,Q,R}→Ø<br>Ø→{O′, P′, Q′, R′}	0.10	0.09
AA	4GT7	A	5HYS	G/I	O→O′<br>Ø→{P′, Q′}	0.02	0.50
OR	1JX6	A	1ZHH	A/B	O→O′<br>{P,Q}→Ø<br>Ø→{P′, Q′}	0.65	0.72
OR	1R42	A	2AJF	A/E	O→P′<br>O→Q′<br>O→R′<br>P→Ø<br>Ø→{O′, S′, T′, U′}	0.14<br>0.73<br>0.14	1.00<br>1.00<br>1.00
OR	1YWH	A	2I9B	E/A	O→Ø<br>P→O′<br>Ø→{P′, Q′, R′, S′}	 0.31	 0.25
OR	1CKL	A	3L89	M/A	O→Ø<br>Ø→O′		
