# Supplementary material for: Quantitative Characterization of the Impact of Protein–Protein Interactions on Ligand–Protein Binding: A Multi-Chain Dynamics Perturbation Analysis Method
Source: Int J Mol Sci. 2024 Aug 23;25(17):9172. doi: 10.3390/ijms25179172 (PMC11394879; doi:10.3390/ijms25179172)
Supplement: Supplementary file 1 [file ijms-25-09172-s001.zip › Table S2-PPI_ZDOCK_on_mcDPA.pdf]

Table S2. Changes in *mc*DPA Predicted clusters for the first 100 ZDOCK conformations of the FTSZ/SULA complex

Complex	Clusters	Overlap	
		Precision	Recall
Complex1	O→Ø		
	P→Ø		
	Q→Q′	0.97	1.00
	R→R′	1.00	1.00
	S→Ø		
	Ø→O′P′S′T′U′V′		
Complex2	O→Ø		
	P→Ø		
	Q→P′	0.97	1.00
	R→O′	0.58	0.94
	S→Ø		
	Ø→Q′		
Complex3	O→Ø		
	P→O′	0.80	0.36
	Q→Q′	1.00	1.00
	R→P′	1.00	1.00
	S→Ø		
	Ø→R′S′		
Complex4	O→Ø		
	P→Ø		
	Q→P′	0.96	1.00
	R→Q′	1.00	0.94
	S→Ø		
	Ø→O′		
Complex5	O→O′	0.29	0.33
	P→Ø′		
	Q→P′	0.95	1.00
	R→Q′	1.00	1.00
	S→Ø′		
	Ø→R′		
Complex6	O→Ø′		
	P→Ø′		
	Q→O′	0.98	1.00
	R→R′	1.00	1.00

	S→Ø′		
	Ø→P′Q′		
Complex7	O→Ø′		
	P→Ø′		
	Q→P′	0.97	1.00
	R→Q′	1.00	1.00
	S→Ø		
	Ø→O′R′		
Complex8	O→Ø′		
	P→Ø′		
	Q→P′	0.89	1.00
	R→O′	0.03	0.11
	R→Q′	0.67	0.56
	S→Ø		
	Ø→R′		
Complex9	O→Ø		
	P→Ø		
	Q→P′	0.81	1.00
	R→Q′	1.00	1.00
	S→Ø		
	Ø→O′R′		
Complex10	O→Ø		
	P→Ø		
	Q→O′	0.98	1.00
	R→Q′	1.00	1.00
	S→Ø		
	Ø→P′R′		
Complex11	O→Ø		
	P→Ø		
	Q→O′	0.94	1.00
	R→Q′	1.00	1.00
	S→Ø		
	Ø→P′R′S′		
Complex12	O→Ø		
	P→Ø		
	Q→P′	0.97	1.00
	R→Q′	1.00	0.94
	S→Ø		
	Ø→O′		

Complex13	O→Ø		
	P→Ø		
	Q→R′	1.00	1.00
	R→O′	0.68	0.67
	S→Ø		
	Ø→P′Q′S′		
Complex14	O→O′	0.20	0.33
	P→Ø		
	Q→R′	1.00	1.00
	R→Q′	1.00	1.00
	S→Ø		
	Ø→P′S′		
Complex15	O→Ø		
	P→Ø		
	Q→P′	0.93	1.00
	R→Q′	1.00	0.94
	S→Ø		
	Ø→O′		
Complex16	O→O′	0.83	1.00
	P→O′	0.75	1.00
	Q→Q′	1.00	1.00
	R→P′	1.00	1.00
	S→Ø		
	Ø→R′		
Complex17	O→Q′	0.09	0.33
	P→Q′	0.04	0.07
	Q→O′	0.93	1.00
	R→P′	1.00	1.00
	S→Ø		
	Ø→R′S′		
Complex18	O→S′	0.29	0.67
	P→Ø		
	Q→P′	0.97	1.00
	R→R′	1.00	0.94
	S→Ø		
	Ø→O′Q′		
Complex19	O→Ø		
	P→Ø		

	Q→R′	1.00	1.00
	R→Q′	1.00	1.00
	S→Ø		
	Ø→O′P′S′		
Complex20	O→P′	0.09	0.33
	P→Ø		
	Q→O′	0.92	1.00
	R→S′	1.00	1.00
	S→Ø		
	Ø→Q′R		
Complex21	O→Ø		
	P→Ø		
	Q→P′	1.00	1.00
	R→Ø		
	S→Ø		
	Ø→O′Q′R′S′		
Complex22	O→Ø		
	P→Ø		
	Q→P′	0.92	1.00
	R→Q′	1.00	1.00
	S→Ø		
	Ø→O′R′S′		
Complex23	O→Ø		
	P→Ø		
	Q→P′	0.97	1.00
	R→Q′	1.00	1.00
	S→Ø		
	Ø→O′R′S′		
Complex24	O→Ø		
	P→Ø		
	Q→P′	0.93	1.00
	R→S′	1.00	1.00
	S→Ø		
	Ø→O′Q′R′		
Complex25	O→Q′	0.18	0.33
	P→Q′	0.18	0.71
	Q→O′	0.96	1.00
	R→P′	1.00	1.00

	S→Ø		
	Ø→R′S′		
Complex26	O→Ø		
	O→Ø		
	P→Ø		
	Q→P′	0.98	1.00
	R→Q′	1.00	1.00
	S→Ø		
	Ø→O′R′		
Complex27	O→R′	0.34	1.00
	P→R′	0.11	0.36
	Q→P′	1.00	1.00
	R→S′	1.00	1.00
	S→Ø		
	Ø→O′Q′T′U′		
Complex28	O→T′	0.67	1.00
	P→Ø		
	Q→O′	0.95	1.00
	R→P′	1.00	1.00
	S→Ø		
	Ø→Q′R′S′		
Complex29	O→O′	0.03	0.33
	P→Ø		
	Q→P′	0.98	1.00
	R→Q′	1.00	1.00
	S→Ø		
	Ø→O′R′S′		
Complex30	O→Ø		
	P→Ø		
	Q→Q′	0.97	1.00
	R→R′	1.00	1.00
	S→Ø		
	Ø→O′P′S′		
Complex31	O→O′	0.10	0.67
	P→O′	0.01	0.07
	Q→Q′	0.89	1.00
	R→R′	1.00	1.00
	S→Ø		
	Ø→P′		

Complex32	O→O′	0.07	0.33
	P→O′	0.04	0.07
	Q→P′	0.98	1.00
	R→R′	1.00	1.00
	S→Ø		
	Ø→Q′		
Complex33	O→O′	0.14	1.00
	P→O′	0.05	0.36
	Q→P′	0.98	1.00
	R→Q′	1.00	1.00
	S→Ø		
	Ø→R′S′		
Complex34	O→O′	0.08	0.33
	P→O′	0.05	0.07
	Q→Q′	1.00	1.00
	R→P′	1.00	1.00
	S→Ø		
	Ø→R′S′		
Complex35	O→Ø		
	P→Ø		
	Q→O′	0.80	1.00
	R→Ø		
	S→Ø		
	Ø→P′Q′R′S′		
Complex36	O→Ø		
	P→Ø		
	Q→Q′	0.98	1.00
	R→P′	1.00	1.00
	S→Ø		
	Ø→O′		
Complex37	O→Ø		
	P→Ø		
	Q→P′	1.00	1.00
	R→Q′	1.00	0.94
	S→Ø		
	Ø→O′		
Complex38	O→Ø		
	P→Ø		

	Q→P′	0.84	1.00
	R→Q′	1.00	1.00
	S→Ø		
	Ø→O′R′		
Complex39	O→Ø		
	P→Ø		
	Q→Q′	1.00	1.00
	R→O′	0.78	0.61
	S→Ø		
	Ø→P′R′S′		
Complex40	O→Ø		
	P→Ø		
	Q→P′	0.94	1.00
	R→O′	0.57	0.78
	S→Ø		
	Ø→Q′R′		
Complex41	O→Ø		
	P→Ø		
	Q→P′	1.00	1.00
	R→Q′	1.00	1.00
	S→Ø		
	Ø→O′R′S′		
Complex42	O→Ø		
	P→Ø		
	Q→R′	1.00	1.00
	R→Q′	1.00	1.00
	S→Ø		
	Ø→O′P′S′		
Complex43	O→Ø		
	P→Ø		
	Q→P′	0.97	1.00
	R→Q′	1.00	0.94
	S→Ø		
	Ø→O′		
Complex44	O→Ø		
	P→Ø		
	Q→P′	0.97	1.00
	R→Q′	1.00	1.00

	S→Ø		
	Ø→O′R′S′		
Complex45	O→Ø		
	P→Ø		
	Q→Q′	1.00	1.00
	R→P′	1.00	0.89
	S→Ø		
	Ø→O′R′		
Complex46	O→T′	0.50	0.33
	P→Ø		
	Q→Q′	1.00	1.00
	R→R′	1.00	1.00
	S→Ø		
	Ø→O′P′S′U′		
Complex47	O→O′	0.05	0.33
	P→O′	0.03	0.07
	Q→Q′	1.00	1.00
	R→P′	1.00	1.00
	S→Ø		
	Ø→R′S′		
Complex48	O→Ø		
	P→Ø		
	Q→S′	1.00	1.00
	R→P′	1.00	1.00
	S→Ø		
	Ø→O′Q′R′T′U′		
Complex49	O→O′	0.02	0.33
	P→Ø		
	Q→P′	0.98	1.00
	R→Q′	1.00	1.00
	S→Ø		
	Ø→R′S′		
Complex50	O→R′	0.10	0.33
	P→Ø		
	Q→O′	0.95	1.00
	R→Q′	1.00	1.00
	S→Ø		
	Ø→P′S′		
Complex51	O→Ø		

	P→Ø		
	Q→O′	0.85	1.00
	R→Q′	1.00	1.00
	S→Ø		
	Ø→P′R′S′T′		
Complex52	O→S′	0.50	0.33
	P→Ø		
	Q→Q′	0.97	1.00
	R→R′	1.00	1.00
	S→Ø		
	Ø→O′P′		
Complex53	O→R′	0.12	0.33
	P→Ø		
	Q→S′	1.00	1.00
	R→P′	1.00	1.00
	S→Ø		
	Ø→O′Q′T′		
Complex54	O→Ø		
	P→S′	1.00	0.64
	Q→P′	0.97	1.00
	R→R′	1.00	1.00
	S→Ø		
	Ø→O′Q′		
Complex55	O→Ø		
	P→P′	0.71	0.57
	P→S′		
	Q→O′	0.97	1.00
	R→R′	1.00	1.00
	S→Ø		
	Ø→Q′		
Complex56	O→Ø		
	P→Ø		
	Q→Q′	0.97	1.00
	R→R′	1.00	1.00
	S→Ø		
	Ø→O′P′S′		
Complex57	O→Ø		
	P→Ø		

	Q→P′	0.97	1.00
	R→Q′	1.00	0.94
	S→Ø		
	Ø→O′R′		
Complex58	O→Ø		
	P→Ø		
	Q→O′	0.84	1.00
	R→R′	1.00	0.89
	S→Ø		
	Ø→P′Q′S′		
Complex59	O→Ø		
	P→Ø		
	Q→Q′	0.97	1.00
	R→R′	1.00	1.00
	S→Ø		
	Ø→O′P′		
Complex60	O→Ø		
	P→Ø		
	Q→Q′	1.00	1.00
	R→S′	1.00	1.00
	S→Ø		
	Ø→O′P′R′		
Complex61	O→O′	0.04	0.33
	O→P′	0.07	0.33
	P→O′	0.52	0.86
	Q→R′	1.00	1.00
	R→S′	1.00	1.00
	S→Ø		
	Ø→Q′T′		
Complex62	O→Ø		
	O→S′	0.25	0.33
	P→Ø		
	Q→P′	1.00	1.00
	R→Q′	1.00	1.00
	S→Ø		
	Ø→O′R′		
Complex63	O→O′	0.36	1.00
	P→Ø		

	Q→P′	0.97	1.00
	R→S′	1.00	1.00
	S→Ø		
	Ø→Q′R′T′		
Complex64	O→Ø		
	P→Ø		
	Q→Q′	1.00	1.00
	R→S′	1.00	0.94
	S→Ø		
	Ø→O′P′R′T′		
Complex65	O→Ø		
	P→Ø		
	Q→Q′	1.00	1.00
	R→O′	0.44	1.00
	S→Ø		
	Ø→P′R′		
Complex66	O→Ø		
	P→Ø		
	Q→R′	0.97	1.00
	R→Q′	1.00	1.00
	S→Ø		
	Ø→O′P′		
Complex67	O→Ø		
	P→Ø		
	Q→Q′	1.00	1.00
	R→R′	1.00	1.00
	S→Ø		
	Ø→O′P′S′		
Complex68	O→S′	0.27	0.33
	P→Ø		
	Q→Q′	1.00	1.00
	R→R′	1.00	1.00
	S→Ø		
	Ø→O′P′		
Complex69	O→Ø		
	P→Ø		
	Q→P′	1.00	1.00
	R→O′	0.82	0.94

	S→Ø		
	Ø→Q′R′S′T′		
Complex70	O→O′	0.21	1.00
	P→O′	0.09	0.29
	Q→Q′	0.97	0.95
	R→P′	1.00	1.00
	S→Ø		
Complex71	O→Ø		
	P→Ø		
	Q→P′	0.88	1.00
	R→R′	1.00	1.00
	S→Ø		
	Ø→O′Q′S′		
Complex72	O→S′	0.29	0.33
	P→S′	0.21	0.21
	Q→P′	1.00	1.00
	R→R′	1.00	1.00
	S→Ø		
	Ø→O′Q′R′T′		
Complex73	O→O′	0.13	1.00
	P→O′	0.07	0.29
	Q→P′	1.00	1.00
	R→Q′	1.00	1.00
	S→Ø		
	Ø→R′		
Complex74	O→Ø		
	P→Ø		
	Q→P′	1.00	1.00
	R→O′	0.17	0.94
	S→Ø		
	Ø→Q′R′S′		
Complex75	O→Ø		
	P→Ø		
	Q→O′	0.85	1.00
	R→Q′	1.00	1.00
	S→Ø		
	Ø→P′R′		
Complex76	O→Ø		

	P→Ø		
	Q→P′	0.58	1.00
	R→O′	0.17	0.72
	S→Ø		
	Ø→Q′R′S′T′U′		
Complex77	O→Ø		
	P→Ø		
	Q→S′	1.00	1.00
	R→Q′	1.00	1.00
	S→Ø		
	Ø→O′P′R′T′		
Complex78	O→Ø		
	P→Ø		
	Q→R′	1.00	1.00
	R→P′	1.00	1.00
	S→Ø		
	Ø→O′Q′S′		
Complex79	O→T′	0.33	0.67
	P→T′	0.67	0.43
	Q→O′	0.88	0.41
	Q→Q′	0.96	0.86
	R→P′	1.00	1.00
	S→Ø		
	Ø→R′S′		
Complex80	O→U′	0.25	0.33
	P→Ø		
	Q→P′	0.98	1.00
	R→Q′	1.00	1.00
	S→Ø		
	Ø→O′R′S′T′V′		
Complex81	O→Ø		
	P→Ø		
	Q→O′	0.87	1.00
	R→S′	1.00	1.00
	S→Ø		
	Ø→P′Q′R′T′		
Complex82	O→Ø		
	P→Ø		

	Q→Q′	0.97	1.00
	R→P′	1.00	1.00
	S→Ø		
	Ø→O′R′		
Complex83	O→Ø		
	P→Ø		
	Q→P′	0.97	1.00
	R→R′	1.00	1.00
	S→Ø		
	Ø→O′Q′S′		
Complex84	O→Ø		
	P→Ø		
	Q→Q′	1.00	1.00
	R→S′	1.00	1.00
	S→Ø		
	Ø→O′P′R′		
Complex85	O→O′	0.09	1.00
	P→O′	0.01	0.07
	Q→Q′	1.00	1.00
	R→R′	1.00	1.00
	S→Ø		
	Ø→P′		
Complex86	O→U′	0.33	0.33
	O→V′	0.67	0.33
	P→U′	0.67	0.21
	Q→O′	0.96	1.00
	R→R′	1.00	1.00
	S→Ø		
	Ø→P′Q′S′T′		
Complex87	O→Ø		
	P→Ø		
	Q→P′	0.87	1.00
	R→Q′	1.00	1.00
	S→Ø		
	Ø→O′		
Complex88	O→R′	0.05	0.33
	P→Ø		
	Q→P′	0.89	1.00

	R→S′	1.00	1.00
	R→Ø		
	S→Ø		
	Ø→O′Q′T′		
Complex89	O→O′	0.10	0.33
	P→O′	0.14	0.07
	Q→P′	0.97	1.00
	R→Q′	1.00	1.00
	S→Ø		
	Ø→R′S′T′		
Complex90	O→O′	0.06	0.33
	P→Ø		
	Q→P′	1.00	1.00
	R→Q′	1.00	1.00
	S→Ø		
	Ø→R′		
Complex91	O→Ø		
	P→Ø		
	Q→Q′	1.00	1.00
	R→P′	1.00	1.00
	S→Ø		
	Ø→O′R′		
Complex92	O→Ø		
	P→Ø		
	Q→R′	1.00	1.00
	R→P′	1.00	1.00
	S→Ø		
	Ø→O′Q′S′		
Complex93	O→Ø		
	P→Ø		
	Q→Q′	0.97	1.00
	R→R′	1.00	1.00
	S→Ø		
	Ø→O′P′S′T′		
Complex94	O→R′	0.03	0.33
	P→Ø		
	Q→S′	0.98	1.00
	R→Q′	1.00	1.00

	S→Ø		
	Ø→O′P′T′		
Complex95	O→O′	0.03	0.33
	P→Ø		
	Q→P′	0.93	1.00
	R→R′	1.00	1.00
	S→Ø		
	Ø→Q′		
Complex96	O→Ø		
	P→Ø		
	Q→O′	0.84	1.00
	R→R′	1.00	1.00
	S→Ø		
	Ø→P′Q′		
Complex97	O→R′	0.10	0.33
	P→Ø		
	Q→P′	1.00	0.95
	R→S′	1.00	1.00
	S→Ø		
	Ø→O′Q′		
Complex98	O→Ø		
	P→Ø		
	Q→O′	0.86	1.00
	R→S′	1.00	1.00
	S→Ø		
	Ø→P′Q′R′		
Complex99	O→S′	0.40	1.00
	P→Ø		
	Q→O′	0.87	1.00
	R→Q′	1.00	0.94
	S→Ø		
	Ø→P′R′		
Complex100	O→Ø		
	P→Ø		
	Q→R′	1.00	1.00
	R→O′	0.50	1.00
	R→P′	1.00	0.39
	R→Q′	1.00	0.33

S→Ø

Ø→S′T′U′
